# High-resolution spatiotemporal weather models for climate studies

**DOI:** 10.1186/1476-072X-7-52

**Published:** 2008-10-08

**Authors:** Michael A Johansson, Gregory E Glass

**Affiliations:** 1 Dengue Branch, Division of Vector-Borne Infectious Diseases, Centers for Disease Control and Prevention, San Juan, PR, USA; 2 W Harry Feinstone Department of Molecular Microbiology and Immunology, Johns Hopkins Bloomberg School of Public Health, Baltimore, MD, USA

## Abstract

**Background:**

Climate may exert a strong influence on health, in particular on vector-borne infectious diseases whose vectors are intrinsically dependent on their environment. Although critical, linking climate variability to health outcomes is a difficult task. For some diseases in some areas, spatially and temporally explicit surveillance data are available, but comparable climate data usually are not. We utilize spatial models and limited weather observations in Puerto Rico to predict weather throughout the island on a scale compatible with the local dengue surveillance system.

**Results:**

We predicted monthly mean maximum temperature, mean minimum temperature, and cumulative precipitation at a resolution of 1,000 meters. Average root mean squared error in cross-validation was 1.24°C for maximum temperature, 1.69°C for minimum temperature, and 62.2 millimeters for precipitation.

**Conclusion:**

We present a methodology for efficient extrapolation of minimal weather observation data to a more meaningful geographical scale. This analysis will feed downstream studies of climatic effects on dengue transmission in Puerto Rico. Additionally, we utilize conditional simulation so that model error may be robustly passed to future analyses.

## Background

Transmission of many infectious diseases is conditioned by the environment. Arthropod-borne diseases are particularly susceptible to climatic influence because transmission is reliant on ectothermic vectors that depend on temperature and often precipitation for development and survival [1] and efficiency as vectors [2]. While many regions of the world have developed systems for high-resolution spatiotemporal disease and sometimes vector surveillance, comparable climate data is rarely available.

Our particular interest is the influence of climate on dengue transmission in Puerto Rico. Dengue viruses cause severe morbidity and occasional mortality in people who become infected. Globally, hundreds of thousands of cases are reported annually to the World Health Organization, including tens of thousands of deaths [3]. The viruses are most often transmitted by a single species of mosquito, *Aedes aegypti*, that lives and breeds in close association with people [4]. Both temperature and precipitation are thought to have significant impacts on dengue transmission because of their effects on the life cycle and transmission potential of *Ae. aegypti *[5-9].

**Figure 1:**
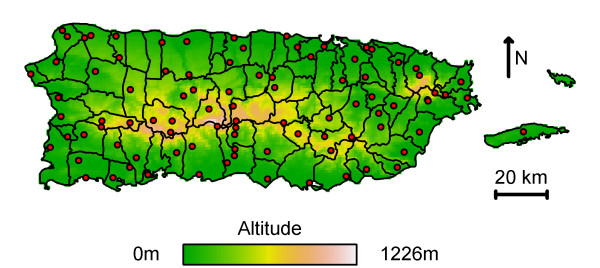
**Puerto Rico COOP weather stations**. Weather stations (red) on a digital elevation model. Municipal borders are shown in black.

The Puerto Rico Department of Health and the U.S. Centers for Disease Control have collected data on reported cases since the 1970s and classified them spatially to the municipal scale based on the patient's residence. Although 76 of Puerto Rico's 78 municipalities are located on a single island 180 km long and 65 km wide, climate varies substantially due to a central mountain range (Figure 1). Most weather systems approach Puerto Rico from the Northeast so northern and mountainous areas are very wet in contrast to the dry southern areas where the air has lost moisture during adiabatic cooling as it passes over the mountains. There is also a North-South trend in temperature; the North is warm, the central mountains, cooler, and the South, hot. This climate variation, combined with the spatial resolution of dengue data, provides a strong framework for assessing the relationship between weather and dengue incidence empirically using longitudinal analysis by exploiting both spatial and temporal heterogeneity. What is lacking is weather data on the same spatial scale as the dengue data.

Weather is most accurately observed directly at weather stations. Throughout Puerto Rico, 92 stations reported weather observations at some point during our study period. Though the accuracy, temporal resolution, and temporal coverage of these measurements are high, the observations are made at specific geographic points which do not represent the entire island. Moreover, the spatial distribution of stations is neither uniform nor constant through time, with as few as 18 stations reporting temperature in a given month. Previous studies of the association between weather and dengue have treated the spatial limitations of observed weather data in different ways. Some have used averaging [10,11] or un-described interpolation methods [12] to estimate regional weather based on observations. Others rely on a single site within the study area [13-16]. Still others contain no detail of the spatial characteristics of the weather data used [17-20].

An alternative to direct observation is using remotely sensed data as a proxy for climate [21]. For temperature, this is achieved reasonably accurately by satellite-measured thermal infrared emissivity, a fairly direct measure of the earth's surface temperature. Measuring precipitation is more complex because measurement is more indirect, relying on proxies such as cold cloud duration (CCD) or the Normalized Difference Vegetation Index (NDVI) [21,22]. While CCD in particular appears to perform better than models based on weather observations under certain conditions [22], there are technical limitations to its utility. Remotely sensed data has traditionally required a significant trade-off between temporal resolution, spatial resolution, and spatial coverage. Though technology is improving, these remain important considerations when undertaking any similar analysis. Here we do not assess the accuracy of remotely sensed proxies because no satellites acquired appropriate data for the temporal resolution, spatial resolution, and temporal coverage of our dengue data set.

Without suitable remotely sensed data to augment estimation, we are left with the observational data. While the temporal coverage and resolution of this data is appropriate, optimization of resolution in the spatial domain is critical to maximize the power of downstream analyses. As mentioned above, previous studies of weather and dengue have neglected to develop and test methods to do this. Here we develop and compare dynamic spatial models utilizing weather station observations to predict weather for the rest of the island using important weather-related covariates including latitude, longitude, altitude, slope, and aspect. Latitude and longitude allow the identification of geographic trends such as the North-South gradient in temperature associated with differing solar exposure. Altitude is an important determinant of temperature because air pressure decreases with increasing altitude. As pressure decreases, air expands, so that heat is adiabatically dispersed. Heat dispersion causes temperature to decrease, increasing water vapor condensation such that clouds form and produce precipitation. While wind moving up a mountain cools and releases precipitation, downsloping wind on the leeward side is drier due to the loss of water vapor and warms as air pressure increases. Slope and aspect (the direction of slope) are critical components of this process, determining how fast warming and cooling occur and where the effects are present geographically.

We analyze three different model types: linear regression, traditional universal kriging, and Bayesian universal kriging (hereto referred to strictly as Bayesian kriging). Linear regression allows prediction for unknown locations based on covariate characteristics. Universal kriging assumes that these covariates do not account for all of the spatial covariance [23]. A spatial covariance structure is therefore fitted to describe the tendency for the observations at proximal sites to be more similar than those further away. In this way, prediction for a given site not only uses the covariate information but also weighted observations from nearby sites. Bayesian kriging is a more computationally intensive alternative to traditional kriging which more conservatively estimates model parameter distributions. An alternative approach to incorporate spatial information into a regression model is spatial smoothing using thin-plate splines [22,24,25]. Though similar, kriging more naturally predicts outcomes at sites which are far from actual observations because it applies little or no observational knowledge to their estimation. In contrast, predictions using thin-plate splines must follow the smoothed surface [26]. Thin-plates splines can thus lead to underestimation of prediction error. Because the intent is to use the resultant model to model the downstream effects of weather on dengue transmission, careful consideration of the estimation error is paramount. To further this aim, we use conditional simulation to produce sets of model-simulated weather outcomes to preserve the model covariance and error in the next stage of analysis.

In the current paper we focus explicitly on the methodology for estimating weather throughout Puerto Rico on scales consistent with dengue surveillance data. We pay particular attention to model development and assessment with the aim of thoroughly describing the methodology such that it may be used in other settings where different spatial and temporal scales may be relevant. Analysis of the relationship between temperature, precipitation, and dengue transmission in Puerto Rico will be published subsequently.

## Results and discussion

### Covariates

We first investigated the selection of month-specific covariates for the prediction of maximum temperature (T_*max*_), minimum temperature (T_*min*_), and precipitation. Altitude entered into every T_*max *_model and longitude, latitude, and slope were accepted as covariates in 42%, 28%, and 13% of the 252 monthly models, respectively (Table 1). For the T_*min *_models, altitude was again universally included. Longitude (15%) and slope (7%) were the other frequently repeated covariates. Altitude was an anticipated covariate because of altitudinal adiabatic cooling. Latitude was expected to be important due to gradient exposure to solar radiation. However, the more frequent inclusion of longitude in models (42 vs. 28%), suggests that the direction of the prevailing winds may be a more important influence in Puerto Rico. Because latitude and longitude create a grid over the surface, they allow the identification of directional trends such as the cooling and warming that occurs as air moves up and down slopes. Thus the dominant factors are altitude and directionality as represented by latitude and/or longitude. Slope may serve as an additional modifier of the directional and altitudinal effects for transitional areas. Secondary covariates were less frequent in the T_*min *_models. T_*min *_reflects night-time temperature which is influenced by both the heat capacity of physical surfaces and changes in advection relative to the structure of those surfaces [27]. Specific surface characteristics were not included in the models.

**Table 1:** Covariate inclusion frequency

	Latitude	Longitude	Altitude	Slope	Aspect
	n (%)	n (%)	n (%)	n (%)	n (%)

T_*max*_	71 (28)	107 (42)	252 (100)	32 (13)	3 (1)
T_*min*_	0 (0)	38 (15)	252 (100)	17 (7)	6 (2)
Precipitation	141 (56)	113 (45)	163 (65)	9 (4)	26 (10)

In precipitation models, the frequently included covariates were altitude (65%), latitude (56%), and longitude (45%, Table 1). The dominance of these covariates is attributable to the movement of air over the island. As hot moist air generated by solar radiation over the ocean moves inland, it encounters physical obstacles in the form of land mass of increasing altitude which causes the moving air to lose pressure, temperature, and then moisture, in the form of rainfall, as it rises. Altitude is an important component of this effect, but directionality is also critical as the air on the leeward side of any mountains is drier due to already having passed over the mountains [28]. The fact that these covariates were not universal likely relates to the highly focal nature of precipitation. While the covariates considered here allow determination of overall trends fairly well, they do not consider small-scale geography such as small hills or lakes that alter precipitation patterns on a more local scale.

### Model selection

**Figure 2:**
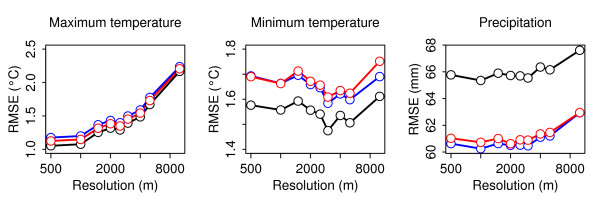
**Cross-validation error by resolution**. Mean RMSE of linear regression (black), universal kriging (blue), and Bayesian kriging (red) models for T_*max *_(A), T_*min *_(B), and precipitation (C). Mean RMSE is significantly different between models at all resolutions for T_*max *_(Bonferroni-corrected, paired *t *test, *α *= 0.05). T_*min *_mean RMSE is significantly lower for the linear regression model at all resolutions. Universal and Bayesian kriging models are significantly different at 3,000, 5,000, and 10,000 meters. Precipitation mean RMSE was significantly higher for linear regression models than for universal or Bayesian kriging models, which were not significantly different at any resolution.

We used linear regression, universal kriging, and Bayesian kriging to predict monthly mean observations for island-wide grids of various resolutions. Assessing the fit of each model is problematic because the limited number of observations makes it desirable to use as many observations as possible for model fitting. For this reason we use cross-validation; each observation is predicted by a model fitted with all observations except the one to be predicted [26]. Generally, removal of one point has little influence on the model parameters, so the parameters are fitted using the entire data set and predictions are made using the appropriate covariates and the spatial covariance model applied to observations at the rest of the sites. For kriging, unlike linear regression, this provides an important comparison because kriging fits the actual observations rather than predicting them solely based upon the model. Though this is computationally efficient, more conservative estimates of error consistent for both regression and kriging are made by completely reparameterizing the model for each observation. We initially take the more efficient approach, measuring prediction error as the root mean square error (RMSE) of observations compared to predictions for all stations at all time points (Figure 2).

Allowing spatial covariation in the temperature models decreased the fit of the models; the simple linear regression model had significantly lower mean efficient cross-validation RMSE at all resolutions compared to the universal kriging or Bayesian kriging models for both T_*max *_and T_*min *_(Figure 2a,b). Although temperature observations do correlate spatially throughout Puerto Rico, most of this variation is accounted for by covariates. Significant spatial structure may be present at some times, but the overall spatial component is minimal so we select the linear regression model for temperature.

The precipitation models, in contrast, contained significant residual spatial variance in the linear regression model as evidenced by the reduced RMSE of universal and Bayesian kriging models (Figure 2c). Of the spatial models, Bayesian kriging produced significantly lower error under the most stringent cross-validation criteria (Table 2).

**Table 2:** Cross-validation error

	Linear regression	Universal kriging	Bayesian kriging
	RMSE (range)	RMSE (range)	RMSE (range)

T_*max *_(°C)	1.24 (0.71–2.13)	1.25* (0.74–2.20)	1.23 (0.76–2.39)
T_*min *_(°C)	1.69* (0.77–3.15)	1.71* (0.77–3.15)	1.83* (0.88–3.16)
Precipitation (mm)	69.2* (13.4–210.3)	65.8* (13.4–210.3)	62.2* (13.4–194.6)

*models significantly different by Bonferroni-corrected paired *t *test (*α *= 0.5)

### Model resolution

The resolution of the model must balance the accuracy and utility of high resolution with the covariate smoothing effects of low resolution. Because we used cross-validation to test the model, we expect the models to perform better at high resolutions where the focus over which geographic features are smoothed is much smaller and thus may be closer to those of the actual station. However, slight changes in grid resolution may increase or decrease the similarity between the characteristics of the prediction pixel and the observation site, so there may be significant flux in the relationship at small intervals.

As expected, T_*max *_models exhibited increased error with decreased resolution (Figure 2). This is likely the effect of decreased accuracy due to smoothing as pixel size increases. T_*min *_models generally had the opposite trend with error decreasing between 500 and 1,000 meters and again, more drastically, between 1,500 and 3,000 meters. This suggests that the determinants of T_*min *_occur over larger areas so that smoothing enhances prediction even as it reduces the specificity of local covariates. The local minima present at 1,000 and 3,000 meter resolutions in the T_*min *_model also occur in the precipitation model. The regular occurrence of minima at 1,000 and 3,000 meters suggests that these are the most appropriate resolutions for this data set. Because the 1,000 meter resolution was the minima in precipitation models, close to the minima in T_*max *_models, a local minima in T_*min *_models, and provides a relatively fine resolution when compared to the smallest administrative area relevant to disease surveillance (approximately 12.5 km^2^), we select it for our final models.

### Model fit

**Figure 3:**
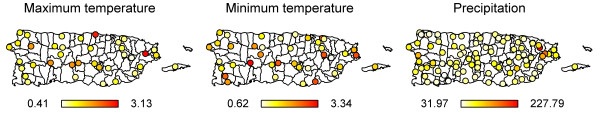
**Cross-validation error by station location**. Mean RMSE for T_*max *_(linear model, °C), T_*min *_(linear model, °C), and precipitation (Bayesian kriging model, mm) at 1,000 meter resolution.

**Figure 4:**
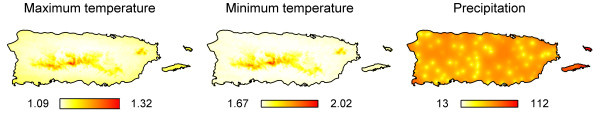
**Prediction error by location**. Mean standard deviation for T*max *(linear model, °C), T_*min *_(linear model, °C), and precipitation (Bayesian kriging model, mm) predictions at 1,000 meter resolution.

Completely refitting each final model for cross-validation at 1000 m resolution, the mean RMSE was 1.24°C for T_*max*_, 1.69°C for T_*min*_, and 62.2 millimeters for precipitation (Table 2). Cross-validation error varied between stations (Figure 3). For T_*max *_and T_*min *_models, increased cross-validation error occurred at higher altitude, likely due to under-representation in observations from those areas. The same problem occurred in the precipitation model, with the majority of error confined to the particularly rainy areas around the El Yunque peak in the northeastern corner of the island. Cross-validation error translates to prediction error (Figure 4). For temperature models, the highest prediction error occurs in the mountainous areas and in coastal locations. Though coastal areas are well represented, directionality is an important covariate component, so as marginal areas they are more susceptible to systemic prediction error.

In the precipitation model, spatial covariance is a critical model parameter. As such, prediction error is very different, principally reflecting station proximity. For example, Culebra, the small island northeast of the mainland, had no observations during this time period. Because of its distance from observation stations, there is very little spatial information to augment covariate information and the prediction error is high. The fact that prediction error increases with distance from observation points is a critical consideration. Kriging models will fit best for areas where observations are relatively close. Though spatial heterogeneity of precipitation is significant even on small scales [29], the cross validation results here show that in Puerto Rico, where the mean distance between proximal weather stations is less than 5 km, additional prediction accuracy is attained by kriging. On a large scale such as the continent of Africa [22], where stations may be hundreds of kilometers apart, kriging may be less useful.

### Conditional simulation

Because we wish to use this model in studies of arbovirus disease transmission, it is critical to have model output that represents the error in the model. Bayesian analytical techniques lend themselves to this purpose because they produce outcome distributions based on simulations that maintain the covariance structure of the model. Simulations can be used in later analyses to account for the possibility of consistent bias in the weather model. The covariance in the simulations preserves model error thus increasing the robustness of downstream analyses.

**Figure 5:**
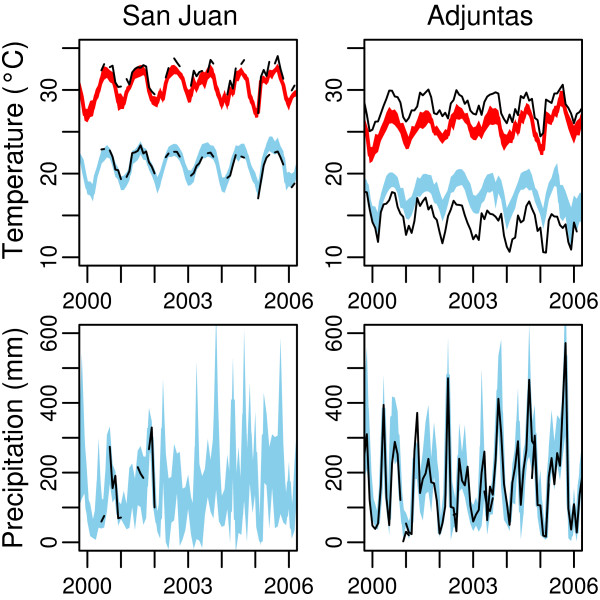
**Temperature and precipitation in San Juan and Adjuntas**. The 95% credible intervals (colors) and actual station observations (black) for San Juan and Adjuntas for 2000 to 2006. T_*max *_(red) and T_*min *_(blue) are predicted maximum and minimum (respectively) mean monthly temperatures for each municipality. Precipitation (light blue) is predicted total monthly precipitation. Black lines are actual observations from stations within each municipality.

Figure 5 shows the mean and 95% credible intervals for T_*max*_, T_*min*_, and precipitation in the municipalities of coastal San Juan and mountainous Adjuntas relative to observations when available. The mean and intervals represent the distribution of estimates for each municipality based on 1,000 simulations of each 1 km^2 ^grid point within the municipality. In San Juan, though observations were not made at all time points, the model predicts those observations based on the stations located in other municipalities. Although there are areas where the model appears to fit the data less accurately, the true time series is susceptible to natural local variation or problems with instrumentation, effects that are reduced by utilizing information collected at other sites. In Adjuntas, observations are more consistent throughout the time series. The model uniformly underestimates T_*max *_and overestimates T_*min*_. This may be due to the specific station location or the fact that Adjuntas is a relatively high-altitude municipality and there are few high-altitude observations.

## Conclusion

We have developed temperature and precipitation models for Puerto Rico that enhance the spatial resolution of raw observational data. The methods presented use basic physical characteristics and spatial information to efficiently predict weather for many locations over an extended time period without making *a priori *assumptions as to what the covariate effects are. Critical to this efficiency is the use of dynamic covariate selection and model fitting, allowing the process to be automated. Because the process is automated, it can be used to convert large datasets of historical weather observations into spatially and temporally pertinent grids. We have developed the current model for dengue studies in Puerto Rico. This purpose drove our target spatial and temporal coverage and resolution. However, the methodology is not limited in this respect. It could equally be applied for smaller or larger areas and shorter or longer time periods. Targets in this respect should consider the biological problem at hand and the ability to analyze the available data with acceptable accuracy.

The raw data used here is discontinuous, spatially-disperse weather observations and globally available altitude data. The ability to convert this data into weather predictions at a meaningful spatiotemporal scale is an invaluable tool for further research. Climate varies drastically in time and space. The impact of this variability on health outcomes must therefore be measured at a relatively fine scale and compared to weather patterns at an equally fine scale. Our model provides a mechanism to address the latter problem. As always, models are limited by the data used to create them, but full utilization of that data, robust cross-validation, and conditional simulation can produce useful predictions with robust error consideration.

## Methods

### Data

Climate observations were obtained from the National Climate Data Center (NCDC) [30]. Excluding a station on the unpopulated island of Mona, between 1986 and 2006, 92 stations reported observed weather (Figure 1). For every month within that time span, 18–33 stations reported 24-hour maximum and minimum temperature and 37–75 stations reported cumulative daily precipitation. Each temperature variable was averaged to a monthly mean to minimize any inconsistencies or missed observations in the data. Cumulative precipitation was also summarized to the monthly scale. Only unaltered first quality data that had not been flagged by NCDC were included.

Altitude, slope, and aspect were derived from the 3 arc-second digital elevation model (DEM) of the Shuttle Radar Topography Mission [31] using Topographic Analysis tools in ERDAS Imagine (version 9.1) [32]. Aspect was recoded categorically as North, East, South, West, or flat. Although we refer to latitude and longitude throughout the paper, the actual analysis used coordinates in the planar State Plane projection for Puerto Rico (NAD 1983, FIPS 5200) to more accurately reflect the spatial landscape of the island.

### Grid generation

Rasters for model output were created as grids with resolutions of 500 to 10,000 meters. This range of resolutions was selected to explore trade-offs between accuracy, computational efficiency, and final resolution. The DEM layer was smoothed to a resolution equivalent to the specified grid size using a square unit filter of size equivalent to the grid resolution using Spatial Modeler in ERDAS Imagine. Covariates for each grid location were extracted from this layer. The filtering ensures that the covariates represent the average for the grid square rather than a point estimate for its center.

### Models

Three different models were assessed: linear regression which ignores the spatial component of the data, and universal and Bayesian kriging models which incorporate spatial structure [23]. The linear models take the form

$$\begin{array}{cc} y_{i}=\beta_{0}+\sum_{p=1}^{P}\beta_{p}x_{i,p}+\epsilon_{i}, & \epsilon \sim N(0,\sigma^{2}), \end{array}$$

for climate variable *y *and covariates *x*_1_...*x*_*P*_, indexed by station location *i*. They are fitted under the assumption that ϵ are independently distributed. Because ϵ may vary geographically, kriging models incorporate a spatial covariance function *S*( ),

$$\begin{array}{cc} y_{i}=\beta_{0}+\sum_{p=1}^{P}\beta_{p}x_{i,p}+S(\phi ,\sigma^{2})+\epsilon_{i}, & \epsilon \sim N(0,\tau^{2}), \end{array}$$

where *ϕ *and *σ*^2 ^parameterize a spatial covariance model. The range, *ϕ*, defines the spatial extent of covariance. The partial sill, *σ*^2^, defines the covariance beyond *ϕ*. Error intrinsic to each location is characterized by the nugget, *τ*^2^, the covariance at zero distance (estimated as a non-zero value due to measurement error and limited sampling at short distances). The statistical package R (version 2.6.0) [33] was used for all models and statistical analyses. Traditional and Bayesian kriging were performed using the package geoR [23]. Code is available from the corresponding author.

All models were developed so that parameterization and prediction occurs exclusively on a monthly scale. For each month, covariates were selected dynamically using stepwise F-tests for inclusion of the covariates latitude, longitude, altitude, slope, and aspect. This approach was taken to allow temporal flexibility because regular and irregular factors, such as hurricanes or changing direction of prevailing winds, may be critical. Another possible approach is to force some variables into the model [22]. We avoided this to reduce the possibility of over fitting the model.

### Model validation

Internal cross-validation was utilized to measure model fit due to the paucity of data. Observations from each station were predicted with data from all the other stations. Initially, for computational efficiency, models were fitted using the complete dataset and prediction was informed by a reduced dataset including all stations except for the prediction site. This omission only affects the kriging models because outcomes for other sites are incorporated into their covariance structure. For selected analyses where greater precision was required, the more computationally demanding method was used in which the model was completely refit for each prediction.

Fit was assessed as the root mean square error (RMSE),

$$\text{RMSE}=\sqrt{\frac{\sum_{i=1}^{n}{\left({\hat{y}}_{j}-y_{i}\right)}^{2}}{n}},$$

where *y*_*i *_is the observation for each station location (*i*) and ${\hat{y}}_{j}$ is the predicted value for the corresponding grid pixel. We use ${\hat{y}}_{j}$ to adjust for predicting gridded values from fixed point observations such that the covariates are not those of the specific point, but rather those of the smoothed grid. RMSE is used because it measures the ultimate goal, accuracy of prediction, rather than the goodness-of-fit of the model. Furthermore, the outcome is intuitively interpreted because the scale is commensurate with the observations. Accuracy for each model and grid resolution was compared using a paired *t *test with Bonferroni correction and was summarized as the mean RMSE over all months.

### Conditional simulation

Final models were used to generate prediction distributions conditioned on the model covariance structure. For the Bayesian model, this is a natural extension as model parameters are assumed to be distributed rather than fixed best estimates. For the linear regression model conditional simulation is accomplished by deriving parameter sets conditioned on the linear model parameter estimates and using these for prediction simulations. This was performed using the R package spBayes [34].

## Competing interests

The authors declare that they have no competing interests.

## Authors' contributions

Both authors participated in study design and manuscript revision. MAJ did the analysis and drafted the manuscript. Both authors read and approved the final manuscript.
